# Predictors of Alcohol Use in Safety-Net Primary Care: Classism, Religiosity, and Race

**DOI:** 10.1155/2020/5916318

**Published:** 2020-06-16

**Authors:** Michael A. Trujillo, Erin R. Smith, Sarah Griffin, Allison B. Williams, Paul B. Perrin, Bruce Rybarczyk

**Affiliations:** ^1^University of California, San Francisco, CA, USA; ^2^Virginia Commonwealth University, Richmond, VA, USA

## Abstract

Class-based discrimination may impact problematic drinking in low-income populations, which may be buffered by personal religiosity. However, little is known how race may impact this association. The purpose of this study was to examine racial differences in the effect of class-based discrimination on problematic drinking as moderated by comfort with God and determine if there were conditional direct effects of class-based discrimination on problematic drinking by race. In this cross-sectional study, participants (*N* = 189) were patients of an urban, safety-net primary care clinic who completed questionnaires assessing experiences of class-based discrimination, attitudes toward God, and alcohol use. Data were collected from 2015 to 2016 and analyzed using the Hayes PROCESS macro. There was a significant main effect for class-based discrimination predicting problematic drinking. Two-way interaction analyses identified a significant comfort with God by race interaction with greater comfort with God associated with less problematic drinking among white but not black respondents. Conditional direct effects showed that experiences of class-based discrimination were associated with problematic drinking at low and moderate but not high levels of comfort with God in black participants, whereas none were observed for white participants. This study provides insight on how personal religiosity, class-based discrimination, and race may intertwine to shape problematic alcohol use in primarily low-income, urban patients. Clinicians' awareness of risk and protective factors, as well as how race tempers the effects of such factors, is vital in providing better care for this population.

## 1. Introduction

Socioeconomic status (SES) has long been known to be a principal determinant of health across a range of outcomes. Individuals with the lowest income and least education, two commonly used indicators of SES, were consistently the least healthy across numerous outcomes, including having more limited activity because of a chronic disease, coronary heart disease, diabetes, obesity, having a lower life expectancy at age 25 [1], and increased alcohol abuse, according to several national data sets [2]. The SES-health link has been observed for centuries and across cultures [3–5] with SES being clearly linked to morbidity and mortality. Race plays an important role in this context with racial/ethnic minorities experiencing elevated rates of disease [6] even at comparable levels of SES [7]. Researchers have posited that low SES may contribute to overall health outcomes through the social environment, including experiences of classism or racism, as well as through coping health behaviors such as substance use [8, 9].

One facet of the social environment that is meaningful in this context is classism. As a system of oppression, classism aims to keep individuals of low SES powerless, while the wealthy remain powerful [10], which can manifest through overt forms of discrimination. Individualistic attributions for poverty (i.e., poor people are responsible for their poverty) are especially prominent [11, 12], suggesting that individuals living in poverty may be more deserving of their social position and therefore more susceptible to individual acts of discrimination based on their SES. Preliminary work has found that the lowest-income individuals are the most likely to experience class-based discrimination and that these experiences are significantly associated with poor health outcomes [13–16]. Experiences of stigma and discrimination are profoundly stressful [17], and the previous findings mirror the more substantial existing literature establishing that discriminatory experiences (e.g., racism) are associated with adverse health outcomes and health behaviors [18–20]. This has a multiplicative effect among racial/ethnic minorities who experience the “double discrimination” of race and class. For instance, black individuals are more likely to experience various forms of discrimination including class-based discrimination [21], resulting in a stronger, adverse influence on the health of black individuals than that of whites of low SES [16].

Heavy alcohol use is one of the lead contributing factors to poor overall health and has been consistently associated with low SES, stress, and experiences with discrimination [20, 22]. Low SES has been associated with frequent binge drinking, alcohol abuse, and alcohol dependence [23, 24] and has been predictive of alcohol use and alcohol problems in longitudinal studies [2] after accounting for gender, age, race/ethnicity, marital status, prior heavy drinking, and present SES [25]. Given the significant exposure to stressors, increased vulnerability to stress faced by individuals of low SES, and chronic strain of economic hardship, drinking may serve as a coping strategy [26, 27]. Furthermore, experiences of discrimination across a range of marginalized social statuses have been associated with a variety of alcohol-related problems [20]. Thus, it is plausible that experiences with class-based discrimination may have a similar impact on alcohol use.

Religiosity has been of interest to researchers as a potentially important factor impacting health and one that may be especially important for individuals exposed to discrimination and those of low SES. Research on the association between religiosity and health is mixed but generally positive with most studies, showing that high levels of religiosity are associated with better health outcomes [28–30]. Religiosity can be defined through an institutional lens (e.g., active attendance of religious services and other activities) [31] as well as by more private forms of involvement (e.g., individual prayer and attitudes about God) [28]. Critically, individuals are likely to overreport attendance of religious services [31], and whereas institutional religiosity is weakly correlated with health, private forms of devotion are more robustly associated with health outcomes [28, 30]. For instance, private forms of religiosity are associated with reduced distress and higher satisfaction with life than institutional forms [28] and may be more accessible to individuals of low SES, given the challenges of transportation to be able to regularly attend services [32]. A meta-analysis of religious coping identified that positive religious coping (e.g., spiritual connection and religious focus) during stress events is associated with positive psychological adjustment [33] and frequency of prayer among worshipers attenuated the effects of stress on well-being, even after accounting for other important factors (e.g., social engagement, healthy lifestyles, and meditation) [34]. Furthermore, religiosity or personal devotion is associated with alcohol abstention [35] and lower levels of alcohol use, abuse, and dependence [29]. Thus, religiosity, and in particular private forms of religiosity, may serve as a protective factor for those who encounter a variety of stressors.

Differences in religiosity by race have also been noted and are likely to impact health. For instance, black individuals report more religious involvement than whites [36, 37]. The “black church” has historically been the primary sociocultural institution whereby black individuals have maintained psychological strength (e.g., self-esteem, optimism, and resilience) in the face of socioeconomic hardships and racial prejudice [38]. Religiosity plays an important role in the way that African-Americans interpret the world, appraise stressors, and construct meaning in times of adversity [39]. Black individuals report more nonorganizational religiosity (e.g., personal devotion) than whites, with black individuals having better physical and mental health through their association with nonorganizational religiosity [40].

Despite the abundance of work linking adverse health outcomes such as problematic drinking to low SES, little research has examined the potential link between experiences of class-based discrimination and drinking. Considering the deficit-based approach to health modeled on marginalized populations such as individuals of low SES and racial minorities, it is imperative to complement these approaches with salutogenic models. In this context, our goal is to examine the health promotive role of private forms of religiosity, especially in the context of race. Therefore, the purpose of this study was to examine the racial differences in the association of class-based discrimination and problematic drinking as moderated by private religiosity in a community sample of patients from an urban, safety-net health clinic. Given the additional stress experienced by black individuals and the importance of the “black church,” a secondary aim was to determine if there were conditional direct effects of class-based discrimination on problematic drinking by race through levels of comfort with God.

## 2. Methods

### 2.1. Participants

Participants (*N* = 210) were adults recruited from the waiting room of an urban, safety-net primary care clinic in Virginia. Inclusion criteria included being at least 18 years of age and a patient of the primary care clinic. Individuals were excluded if they did not meet a minimum score (≥10) on a brief health literacy screener [41]. As the purpose of the current study was interested in differences between black and white individuals, those identifying outside of these racial categories were dropped from the current analyses (*n* = 21). The number of respondents who were not eligible for the study due to low literacy scores was not tracked. The final sample considered the experiences of 189 participants. See Table 1 for participant demographics.

### 2.2. Measures

Participants completed a set of questionnaires assessing experiences of class-based discrimination, attitudes toward God, and alcohol use, as well as researcher-generated demographic items. Race was assessed via one item asking respondents to select a race/ethnicity label that best described them including black/African-American (non-Latino) and white/European-American (non-Latino), among others.

#### 2.2.1. Everyday Discrimination Scale-Short Version (EDS)

Experiences of class-based discrimination were assessed with the 5-item EDS [42], a measure aimed to evaluate broad experiences of discrimination. In the original measure, respondents are asked to respond to the following prompt “In your day-to-day life, how often have any of the following things happened to you?” The original prompt was adapted for the current study to include “because of your income level or social class” at the end of the stem. The EDS has a 6-point response scale ranging from 0 (*never*) to 5 (*almost every day*). A total score is produced by the summation of the five items, with higher scores indicative of greater experiences of class-based discrimination. The internal consistency of the measure has previously been demonstrated (*α* = 0.77) [42]. The current study evidenced good reliability for both the black (*α* = 0.80) and white subsamples (*α* = 0.79).

#### 2.2.2. Attitudes toward God Scale (ATGS)

The ATGS [43] is a 9-item measure that assesses feelings of comfort and positive attitudes toward God and feelings of anger toward God. The items prompt respondents to identify how strongly they currently feel toward the content of a particular item across two domains: comfort with God (e.g., “feel nurtured or cared for by God”) and anger with God (e.g., “feel that God has let you down”). Both domains use an 11-point response scale from 0 (*not at all*) to 10 (*extremely*) with each subscale scored by averaging across items. Higher scores reflect greater comfort or anger with God. For the purpose of this study, only the comfort with God subscale was used in order to examine if this form of religiosity served as a protective factor. The measure has evidenced good internal consistency across both subscales in ethnically diverse samples (*α* range: 0.80–0.96), as well as good construct and discriminant validity [43]. Internal consistency for the black (*α* = 0.94) and white (*α* = 0.92) subsamples was good.

#### 2.2.3. Alcohol Use Disorders Identification Test-Consumption (AUDIT-C)

The AUDIT-C [44] is a 3-item alcohol screen used to identify persons who have active alcohol use disorders (including abuse and dependence) or who are hazardous drinkers. The items assess the frequency of drinking alcohol, the number of drinks consumed on a typical day, and the frequency of drinking six or more drinks on one occasion. The scale is scored on a scale of 0–12 with responses indicating the least amount of drinking for a given item as 0 and the maximum as 4. In men, a score of 4 or more is considered positive for hazardous drinking or the presence of an active alcohol use disorder, while for women, the criterion is a score of 3 or more. The total score was used in the current study with higher scores indicate greater problematic drinking. The AUDIT-C has significant clinical utility and has previously been used in a primary care setting [45], and AUDIT-C has shown excellent psychometric properties in the US general population [46]. It yielded good reliability for both black (*α* = 0.83) and white subsamples in the current study (*α* = 0.78).

### 2.3. Procedure

Participants were recruited from an urban, safety-net clinic focused on providing care for primarily indigent and low-income individuals. Researchers approached individuals by first asking if they were a patient of the clinic and if they were interested in hearing more about a study assessing their needs as a patient. Individuals were told that the purpose of the study was to better understand the current needs of patients as well as additional experiences that might be important for their health. Interested individuals completed a health literacy screener to assess for reading comprehension of medical information. If individuals met the minimum score necessary indicating they had sufficient health literacy to comprehend the survey items and instructions, they completed an institutional-review board-approved informed consent form followed by a survey estimated to take between 30 and 45 minutes. If they were called back by clinic staff while completing their survey, they were asked to briefly stop where they were but could resume once their appointment ended. All eligible participants were compensated with $10 cash upon completion of the survey. Participant recruitment took place between October 2015 and July 2016.

### 2.4. Statistical Analyses

Prior to running the primary analyses, a series of bivariate correlations were conducted by race using SPSS, version 26.0 (IBM, 2019). A single moderated moderation model that included all variables in one step was tested using the Hayes [47] PROCESS macro (model 3) assessing whether comfort with God moderated the relationship between class-based discrimination (EDS) and problematic drinking (AUDIT-C) and whether this moderation was further moderated by race, that is, a three-way interaction. To address the secondary aim, a test of conditional direct effects of the moderation was conducted as a function of race. Tests used 5,000 bootstrap samples. All variables in the model were continuous, except for race, which was treated as categorical with white, non-Hispanic set as the reference group. Comfort with God was stratified into three groups (one standard deviation [SD] below the mean, within one SD from the mean, one SD above the mean) only for the conditional direct effects analysis. To account for gender differences in alcohol use [48], gender was included as a covariate in analyses. All estimates are unstandardized.

## 3. Results

### 3.1. Missing Data

Prior to running the primary analyses, expectation maximization was used to impute missing data using SPSS, version 22.0. Between <1% and 5% of variables in the entire data set had missing data; items querying trauma had the most missing (though not used in the current study). For the current analyses, <3% of variables had missing data and were subsequently imputed. To determine whether the data were missing completely at random prior to imputation, three Little's MCAR tests were conducted on each scale or subscale used. All tests were not significant (*p'*s ≥ 0.128), indicating that the data were missing completely at random and suggesting that multiple imputation was appropriate. Twenty-five iterations were conducted during imputation.

### 3.2. Preliminary Analyses

Normality assumptions were assessed prior to running the primary analyses. All measures met criteria for normality with skewness values ranged from −1.543 to 1.282 and kurtosis values ranged from −0.454 to 1.219. An assessment of the scatterplot for each measurement showed no evidence of outliers. Tolerance, VIF, and Mahalanobis *D*^2^ were used to assess multicollinearity. The value of 0.97 for tolerance and 1.027 for VIF as well as an assessment of Mahalanobis *D*^2^ all indicate the absence of multicollinearity.

#### 3.2.1. Racial Differences

A total of three *t*-tests were conducted on key variables to identify any differences by race. No racial differences were observed for class-based discrimination (*t*(187) = 0.98, *p*=0.327) or for problematic drinking (*t*(187) = −0.06, *p*=0.957). There was a significant difference in comfort with God (*t*(187) = −4.05, *p* < 0.001) with black respondents reporting greater comfort with God (*M* = 8.82, *SD* = 2.41) compared with white respondents (*M* = 7.15, *SD* = 2.97; Table 2).

#### 3.2.2. Correlations by Race

Among black respondents (*n* = 132), EDS was positively associated with AUDIT-C scores and negatively associated with comfort with God (Table 2). Comfort with God was not significantly associated with AUDIT-C scores. Among white respondents (*n* = 57), AUDIT-C scores were negatively associated with comfort with God. Neither Comfort with God nor AUDIT-C scores were associated with the EDS (Table 2).

### 3.3. Primary Analyses

Prior to analyses, a combination of *t*-tests and chi-square analyses was conducted to identify if racial differences existed for a variety of demographic variables (i.e., age, income, education, and employment status) in order to account for them in the primary analyses. No group differences were identified across any variables (*p* ≥ 0.142) and therefore not included in analyses.

**Figure 1:**
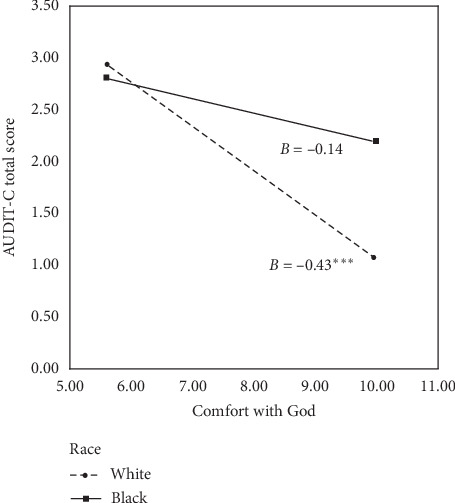
Interaction of comfort with God by race predicting AUDIT-C scores. Note: AUDIT-C = Alcohol Use Disorders Identification Test-Consumption; *B* = unstandardized estimate. ^*∗∗∗*^*p* < 0.001.

The overall model of EDS, comfort with God, and race predicting AUDIT-C scores was significant (*F*(8, 180) = 4.65, *p* < 0.001, *R*^2^ = 0.17), after accounting for gender. There was a significant main effect for EDS (*B* = 0.09, *p*=0.006; Table 3) such that greater EDS scores were associated with greater AUDIT-C scores. The main effect for comfort with God was marginally significant (*B* = −0.16, *p*=0.052), with the main effect for race not reaching the level of significance (*B* = 0.61, *p*=0.193; Table 3). An examination of two-way interactions identified a significant comfort with God × race interaction (*B* = 0.42, *p*=0.009; Figure 1). A simple slopes analysis identified a significant negative association between comfort with God and AUDIT-C scores for white (*B* = −0.43, *p* < 0.001) but not black respondents (*B* = −0.14, *p*=0.168). All other two-way interactions were not significant (*p's* ≥ 0.119). The interaction of EDS × comfort with God × race was marginal but not significant (*B* = −0.04, *p*=0.063).

**Figure 2:**
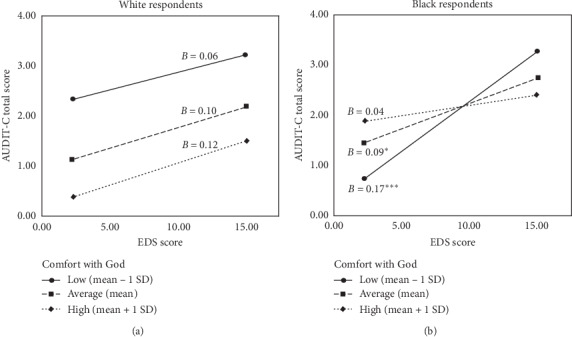
Conditional direct effects of EDS on AUDIT-C scores at levels of comfort with God by race. Note: EDS = Everyday Discrimination Scale; AUDIT-C = Alcohol Use Disorders Identification Test-Consumption; *B* = unstandardized estimate. ^*∗*^*p* < 0.05 and ^*∗∗∗*^*p* < 0.001.

For exploratory purposes, we probed the marginally significant three-way interaction for conditional direct effects of EDS on AUDIT-C scores at levels of comfort with God by race (Table 4). This procedure tests the predictor-criterion relation at low (i.e., 1 SD below the mean), average (i.e., mean), and high (i.e., 1 SD above the mean) levels of comfort with God. Analyses identified a significant conditional interaction of EDS and comfort with God among black [*F*(1, 180) = 6.06, *p*=0.014] but not white [*F*(1, 180) = 0.47, *p*=0.490] respondents. The simple slopes analyses (Table 4) identified that the EDS was not significantly associated with AUDIT-C scores at low, average, or high levels of comfort with God among white respondents (*p's* > 0.151; Figure 2). However, EDS was significantly associated with AUDIT-C scores at low (*B* = 0.17, *p* < 0.001) and average (*B* = 0.09, *p*=0.019) levels of comfort with God but not at high levels (*B* = 0.04, *p*=0.380; Figure 2).

## 4. Discussion

The purpose of this study was to identify if racial differences exist in the association of class-based discrimination and problematic drinking as moderated by positive, private religiosity in a community sample of patients from an urban, safety-net primary care clinic. A probing of the marginally significant three-way interaction showed that experiences of class-based discrimination were associated with problematic drinking at low and moderate but not high levels of comfort with God among black participants, whereas no conditional direct effects were observed for white participants, after accounting for gender. Two-way interaction analyses identified a significant comfort with God by race interaction such that there was a significant negative association for white but not black respondents. There was a significant and positive main effect for class-based discrimination such that more discrimination was associated with greater problematic drinking. Comfort with God exhibited a marginally significant, negative main effect.

It is surprising that comfort with God did not altogether buffer the relationship between class-based discrimination and problematic drinking, given the generally positive influence of personal religiosity on health outcomes [28, 35, 49]; however, additional work has also failed to find significant associations [50], indicating a potentially more complex relationship may be taking place. A probing of the marginally significant three-way interaction found that comfort with God buffers the association between class-based discrimination and problematic drinking but only at the highest levels of comfort with God for black individuals. Comfort with God mitigated the association of class-based discrimination and problematic drinking at all levels for white individuals. While these results are generally in line with work identifying private forms of religiosity with less problematic drinking [29], they are interesting in light of previous research identifying African-Americans as being more religiously involved [36, 37] and especially considering that black respondents in the current sample also reported greater comfort with God. However, black individuals also have to contend with a system of racism that further marginalizes them and makes them susceptible to experiences of racial discrimination in addition to that based on class. For instance, black individuals are more likely to report racial discrimination than whites [51], which impacts alcohol use [52, 53] and has unique adverse effects on general health beyond nonracial discrimination for black people [54]. It is possible that individuals who report lower levels of comfort with God are also unable to buffer the impact of racial discrimination that may subsequently impact health. As racial discrimination was not assessed in the current study, future work should aim to disentangle the effects of race- and class-based discrimination and its association with alcohol use.

For white individuals, comfort with God serves as a protective factor in the relationship between class-based discrimination and problematic drinking. These results are likely driven by the significant negative association between comfort with God and problematic drinking among white but not black respondents evidenced by the racial differences in the bivariate correlations. For instance, prior work found that intrinsic religiosity (i.e., importance of spiritual beliefs), a strong correlate of comfort with God [43], was associated with reduced alcohol use among white but not black respondents [37]. Additionally, religious denomination may play an important role in alcohol use [55] with individuals from conservative Protestant denominations having a stronger moral message that prohibits alcohol use [56]. As religious denomination was not assessed, future work should aim to include this in relation to alcohol use.

When these results are considered in the context of the significant two-way interaction, it also highlights differences in how private forms of religiosity may be utilized. That comfort with God was not associated with problematic drinking among black individuals compared with the significant negative association among white individuals supports prior literature evidencing religiosity as an important cultural resource among African-Americans but particularly during times of adversity [39]. Black individuals are more likely to use religion as a coping mechanism in times of crisis and physical suffering [57] and are specifically more likely to use a personal devotion form of religiosity as a coping strategy across a range of problems than whites [58, 59]. This is evident by the significant negative correlation between comfort with God and class-based discrimination evident among black but not white individuals in the current sample. In this way, black individuals may be more likely to draw upon their belief in being comforted by God as a source of strength to help them through stressful situations, such as class-based discrimination, that may lead to reduced problematic drinking. This likely explains why comfort with God was associated with reduced problematic alcohol in the conditional direct effects but not in the two-way interaction.

It is also possible that the beneficial effects of religiosity may be in part due to social support and access to a social network. One possibility is that black respondents who reported high compared with average or low levels of comfort with God may also be receiving more social support or have access to a larger social network, which impacts alcohol use [60]. Religiosity incorporates both psychological and instrumental social support for religiously oriented individuals but especially for African-Americans [39, 61], with individuals of low SES providing and receiving more support than their higher SES counterparts [62]. As social support or social network was not assessed in the current study, future work should aim to examine their role for alcohol use.

That class-based discrimination exhibited a significant main effect even when accounting for the multiple interactions underlies its importance for health. Though previous work on the association of class-based discrimination and alcohol use is limited, the current results are in line with the broader discrimination literature outlining a positive association with greater alcohol use and abuse [52]. These results point to including class-based discrimination in broader discussions of discrimination and alcohol use problems and contribute to the paucity of work linking class-based discrimination and health. That prior work found no association between class-based discrimination and alcohol use [13] may be due to differences in the measurement of classism (e.g., binary versus continuous). For instance, Simons et al. [13] analyzed perceived classism as a binary predictor, whereas the current study treated class-based discrimination as a continuous variable. This difference may have led to a difference in power [63] to detect a significant association leading to contrasting results.

Additionally, neither race nor comfort with God interacted with class-based discrimination in its association with problematic drinking, which appears to contradict prior work. Despite the presence of racial differences in the perception of discrimination [21, 64], the results of the current study indicated that black and white respondents experience comparable rates of class-based discrimination, at least in the current sample. Prior studies on racial differences have primarily focused on perceptions of class-based discrimination from health care providers [21] and not in everyday domains. Future work should aim to examine class-based discrimination more broadly to identify whether racial differences exist outside of the health care field.

### 4.1. Implications

The present study has important implications for health care and policy. First, this study demonstrates the potential role that class-based discrimination may have for problematic drinking, in general, but especially for black individuals with low and moderate levels of comfort with God. Broadly, clinicians should be attuned to the potential harms of class-based discrimination and that black patients, especially those who do not have the protective factor of faith, may be more vulnerable. Additionally, an assessment of religiosity at intake may be beneficial. Given that a full assessment at intake may be unrealistic, patients may be probed by asking simple questions such as “Do you regularly engage in prayer?” Clinicians may be trained to assess which individuals might benefit from strengthening their comfort with God and refer them to local faith communities, with a particular focus on black churches for black patients. It would therefore behoove clinics to establish partnerships with local faith communities. Patients may be provided with information on the presence and support of these communities on a local resources page that can be given to all patients at the completion of a new appointment as well as on a community board that all patients have access to. As it is common for clinics to regularly offer patients information on resources, this addition would not be intrusive or burdensome to clinic staff.

These results also have important policy implications. Considering that the majority of the samples lived in poverty (at least 82.4% making less than $9,999), were unemployed or on public assistance (82.8%), and either did not have health insurance or had difficulty paying for their care (86.1%), policies that advocate for increasing resources to improve housing, financial stability, and health care coverage are necessary to care for those most vulnerable in society. Given that alcohol use may be used as a coping mechanism to address stress for individuals living in poverty [26, 27] and that classism is an ingrained ideology that is resistant to change, policies that aim to lift people out of their marginalized social positions not only improves their health but can also reduce exposure to class-based discrimination.

### 4.2. Limitations

The current study has limitations that warrant caution. The cross-sectional nature of the study precludes causal interpretations. Although the interpretation of the results is such that class-based discrimination leads to greater alcohol use, the opposite direction may also be operating, that is, greater alcohol use may lead to homelessness or reduced income, which would then expose a person to class-based discrimination. Future studies should examine these associations using cross-lagged panel designs to more directly infer causality. Additionally, the smaller white subsample may have led to a reduction in power, obfuscating our ability to detect significant effects. Religious denomination was also not assessed, which may impact how religiosity is associated with drinking behavior. The construct of comfort with God could differ dramatically as a function of what religious denomination a person adheres to. Future research should more thoroughly assess participant religiosity and be conducted in a larger sample. Finally, the current study also did not assess for the types of services that patients were seeking including substance use treatment, which may impact the series of relationships presented herein. Although behavioral health treatment was offered at the particular clinic where participants were recruited, it is unknown whether the use of behavioral health services impacted alcohol use.

## 5. Conclusions

A moderated moderation analysis examined the effect of class-based discrimination on problematic drinking, as moderated by comfort with God and race in a sample of patients from an urban, safety-net primary care clinic. Problematic drinking was significantly associated with class-based discrimination and was marginally associated with comfort with God. A significant race by comfort with God interaction was qualified by a marginally significant three-way interaction between class-based discrimination, comfort with God, and race. Class-based discrimination may be more important for problematic drinking among black than white individuals, particularly among those who report less comfort with God. Future work should further aim to understand the complex nature by which class-based discrimination interacts with race in its association with alcohol use in community samples. This study provides valuable insights into how private forms of religiosity, class-based discrimination, and race intertwine to shape excessive alcohol use in low-income, urban patients. Clinicians' awareness of risk and protective factors is vital to providing better care for this population.

## Figures and Tables

**Table 1:** Demographics of the study sample (*N* = 189).

Variable	Black subsample (%)	White subsample (%)	Total sample (%)
Age, M (SD)	45.39 (11.00)	43.67 (12.66)	44.97 (11.53)
Female	43.3	36.8	58.7
Sexual orientation			
Heterosexual	85.2	89.5	87.7
Gay/lesbian	6.3	5.3	5.4
Bisexual	7.8	3.5	5.9
Queer	0.8	1.8	1.0
Education			
Middle school/junior high	9.0	8.8	9.0
High school	57.9	40.4	52.7
Some college (no degree)	22.6	29.8	24.5
2-year/technical degree	3.8	3.5	3.7
4-year college degree or higher	6.8	17.6	10.1
Current employment status			
Unemployed	66.4	66.7	66.1
On public assistance	16.0	17.5	16.7
Full-time	5.3	1.8	4.3
Part-time	9.9	8.8	9.7
Homemaker, student, or retired	2.3	5.4	3.2
Past year income or public assistance received			
$0-$4,999	70.7	66.7	69.1
$5,000-$9,999	14.3	10.5	13.3
$10,000-$14,999	6.0	8.8	6.9
$15,000-$19,999	3.8	7.0	4.8
$20,000-$24,999	1.5	3.5	2.1
$25,000 or greater	3.8	3.5	3.7
Currently without permanent housing	61.7	63.2	61.7
Current length of time without permanent housing			
Less than 1 month	28.7	15.0	25.0
1–3 months	20.2.	25.0	22.0
3–6 months	10.6	12.5	11.4
6–9 months	9.6	15.0	9.8
12 months or more	30.8	32.5	31.3
Has previously been without permanent housing	51.9	47.4	51.1
Currently has health insurance	54.5	50.9	54.0
Has health insurance but is unable to pay for care	44.2	31.5	40.1
Meets criteria for hazardous drinking	29.8	30.3	30.0

M = mean; SD = standard deviation.

**Table 2:** Pearson product-moment correlation coefficient matrix, means, and standard deviations by race.

	1	2	3
1. EDS	—	0.074	0.075
2. AUDIT-C	**0.242** ^*∗∗*^	—	−0.514^*∗∗∗*^
3. Comfort with God	**−0.257** ^*∗∗*^	**−0.113**	—
Mean (SD), black (NL)	8.13 (6.41)	2.33 (3.01)	8.82 (2.41)^a^
Mean (SD), white (NL)	9.11 (5.92)	2.31 (2.66)	7.15 (2.97)^a^

EDS = Everyday Discrimination Scale; AUDIT-C = Alcohol Use Disorders Identification Test-Consumption; SD = standard deviation; NL = non-Latino. Bold values reflect correlations among black respondents. Nonshaded values above the diagonal reflect correlations among white respondents. ^a^Significantly different from each other at *p* < 0.001. ^*∗∗*^*p* < 0.01 and ^*∗∗∗*^*p* < 0.001.

**Table 3:** Model summary for the association between EDS, comfort with God, and race predicting AUDIT-C scores.

Predictor	Unstandardized estimates (SE)	95% bootstrap confidence interval
EDS	0.09 (0.03)^*∗∗*^	[0.03, 0.16]
Comfort with God	−0.16 (0.08)^+^	[−0.32, 0.00]
Race^a^	0.61 (0.46)	[−0.31, 1.52]
EDS × comfort with God	−0.02 (0.01)	[−0.04, 0.00]
EDS × race	−0.01 (0.08)	[−0.16, 0.15]
Comfort with God × race	0.42 (0.16)^*∗∗*^	[0.11, 0.74]
EDS × comfort with God × race	−0.04 (0.02)^+^	[−0.09, 0.00]
Gender^b^	−0.97 (0.41)^*∗*^	[−1.79, −0.15]
*R* ^2^	0.17	

EDS = Everyday Discrimination Scale; AUDIT-C = Alcohol Use Disorders Identification Test-Consumption; SE = standard estimate. Estimate values are unstandardized betas. ^a^Reference category is white, non-Hispanic. ^b^Reference category is man. ^+^*p* < 0.07, ^*∗*^*p* < 0.05, and ^*∗∗*^*p* < 0.01.

**Table 4:** Conditional direct effects by race of EDS on AUDIT-C total score moderated by comfort with God.

Comfort with God	Unstandardized estimates (SE)	95% bootstrap confidence interval
White		
Low	0.06 (0.07)	[−0.08, 0.20]
Average	0.10 (0.07)	[−0.04, 0.23]
High	0.12 (0.09)	[−0.05, 0.30]

Black		
Low	0.17 (0.05)^*∗∗∗*^	[0.08, 0.27]
Average	0.09 (0.04)^*∗*^	[0.02, 0.17]
High	0.04 (0.05)	[−0.05, 0.13]

EDS = Everyday Discrimination Scale; AUDIT-C = Alcohol Use Disorders Identification Test-Consumption; Low = mean minus one standard deviation (5.62); average = mean (8.31); High = mean plus one standard deviation (10.00); 5,000 bootstrap samples. Estimate values are unstandardized betas. ^*∗*^*p* < 0.05 and ^*∗∗∗*^*p* < 0.001.

## Data Availability

The data used to support the findings of this study are available from the corresponding author upon request.
